# Exploring trust dynamics in health information systems: the impact of patients’ health conditions on information source preferences

**DOI:** 10.3389/fpubh.2024.1478502

**Published:** 2024-11-22

**Authors:** Mingming Song, Joel Elson, Tin Nguyen, Sharon Obasi, John Pintar, Dhundy Bastola

**Affiliations:** ^1^School of Interdisciplinary Informatics, University of Nebraska Omaha, Omaha, NE, United States; ^2^College of Arts and Sciences, University of Nebraska Omaha, Omaha, NE, United States; ^3^Department of Counseling, School Psychology and Family Science, University of Nebraska at Kearney, Kearney, NE, United States

**Keywords:** health information system, trust preferences, information sources, symptom intensity, disease type, cognitive trust, behavioral trust

## Abstract

**Introduction:**

Health information systems (HISs) should provide accessible and high-quality information to patients. However, the challenge lies in understanding patients’ trust preferences for health information. This study explores how different information sources (e.g., online platforms, interpersonal sources) are trusted under varying health conditions, focusing on symptom intensity and disease type.

**Methods:**

Using a 2 × 2 × 4 between-subject design, 243 participants from a US college were presented with vignettes of acute or chronic diseases with varying symptom intensities and information sources. Participants rated their trust levels, including both cognitive and behavioral trust, in the health information and recommendations provided by one of the information sources, which was randomly assigned. Logistic regression and ANOVA were employed for the statistical analysis.

**Results:**

The analysis results revealed that trust is generally higher for interpersonal sources like doctors and family/friends compared to online sources like WebMD and Wikipedia when patients are making health decisions. Doctors are the most trusted source during health-related decision making. However, there are no significant differences in cognitive trust among interpersonal sources or among online sources. Furthermore, symptom intensity and disease type did not significantly alter trust levels across various information sources. These findings suggest that people prefer professional medical advice regardless of their health conditions.

**Discussion:**

The study highlights the need for HIS to incorporate features that provide “doctor-verified” information and promote interactive engagement to enhance patients’ trust in information source. Additionally, it distinguishes between cognitive and behavioral trust, revealing distinct trust patterns that can inform the strategic development of HIS for varied health conditions. Understanding these trust dynamics can inform the design of effective, patient-centered HIS that better support health education, information seeking, and decision-making.

## 1 Introduction

Health information is essential in the healthcare journey. Health information systems (HISs) are not only designed as computing systems to capture, store, and manage patients’ data, but they can also be used to provide accessible and high-quality information to patients (1–3). However, a challenge to designing effective HIS lies in people’s trust preferences for information relating to their health concerns. Generally, people seek and trust health-related information from different sources (e.g., online platforms, personal connections, healthcare professionals) (4). While some rely on online sources like WebMD, others may prefer traditional channels, such as healthcare providers or recommendations from friends or family (5–7). Some studies suggest that people rely on online information sources in the short-term and ultimately place trust in their doctors (6, 8). Other evidence points to a more nuanced picture regarding who trusts which sources of health information and why (7). To design an effective HIS, it is essential to understand how humans form trust in various sources of knowledge. This requires identifying the factors that influence when and why people trust certain sources. While previous studies have explored trust dynamics in health information sources, our study extends this research by differentiating between cognitive and behavioral trust and examining their influence under varying health conditions.

Elements of a health condition, such as symptom intensity and acuteness, can affect which sources of health-related information people trust and rely upon. Research shows that the intensity and severity of physical ailments can alter people’s information-seeking behaviors (9, 10). For instance, patients with more severe conditions may prefer to directly communicate with their doctors due to the complexity of their medical needs and the necessity for timely care (11, 12). The severity of a disease can be positively correlated with the frequency of online medical searches (9). This may be due to patients’ need for comprehensive information that provides detailed insights (13). This suggests that the characteristics of health condition itself might play a critical role in shaping individual reliance on and trust in various information sources. Therefore, to better understand this relationship of trust on the source of information, we consider two key aspects: symptom intensity and disease severity in the present study. Here the symptom intensity refers to the level or magnitude of symptoms experienced by patients, such as pain, fatigue, or aches (14). This measure helps quantify how intense the symptoms are, ranging from low to high. Severity encompasses a broader evaluation of a patient’s health condition. It includes not only the intensity of symptoms but also how patients perceive their overall health status and consider factors such as disease type and overall impact of the condition on their daily lives (15). Therefore, in this study, symptom intensity and disease type are measured as two independent factors.

This study aims to understand how these two factors impact patients’ willingness to accept or trust health-related information from different sources. Specifically, we investigate whether trust in the same health-related suggestions varies depending on the information source, symptom intensity, and disease type. By examining these relationships, we aim to gain nuanced understanding of how people evaluate information sources and the potential factors that influence their healthcare decisions. This knowledge will inform strategies to enhance HIS to better support health education, information seeking, and informed decision-making.

HIS should be designed with an understanding of people’ trust variance because digital tools for health education, data management, and individual support process complex relations between different pieces of information (16, 17). By understanding the factors that affect trust in health information from diverse sources, this study aims to inform the design of patient-centered HIS that educates and assists individuals as they navigate health-related decisions. The challenge lies in ensuring that these systems are reliable, credible, and aligned with patients’ trust preferences, so that they effectively meet the informational needs of diverse patients (18, 19). For instance, if it is established that people strongly prefer health information from doctors over other online sources, then the design of the system should prioritize doctor-provided information to enhance patients’ utilization and adherence. Alternatively, if people show preferences toward non-expert interpersonal sources, this points to a need to implement high-quality health information through interactive channels (e.g., moderated forums with specialized, well-informed moderators). The aim of this study is to understand such trust preferences and contribute to the broader discussion on how health informatics can be optimized to meet the complex and varied needs of people in accessing and trusting health information.

To explore trust dynamics, previous studies have employed survey methodologies to examine patients’ trust in health information across various sources (20). While these surveys were effective in identifying correlations or associations between factors such as demographic information of participants and their trust level, they are limited in establishing causality (21). To overcome this limitation, we propose an alternative approach using experimental vignette studies. By manipulating contextual factors and testing our research through the creation of vignettes, we can explore the factors that influence trust in different information sources. This approach enables us to overcome participant limitations and draw causal inferences that directly inform the design of patient-centered HIS.

## 2 Background and hypotheses development

In the rapidly evolving digital health ecosystem, patients are exposed to a diverse array of health information sources that convey varying levels of expertise, reliability, and trustworthiness. The construction of trust on health information is influenced by multiple factors, including the context in which the information is presented, the relationship between the patient and the information provider, and the perceived credibility of the source (22, 23). With the advancements in technology and the widespread availability of online information, patients now have access to a broader range of health information sources from which to draw. To better understand how these different sources compare, we examine interpersonal sources such as friends, family, and doctors, alongside online health information sources like Wikipedia and WebMD. This comparative analysis aims to evaluate the characteristics and trust levels associated with each type of information source.

Review of literature suggests that individuals seeking health-related information tend to trust sources that effectively convey quality features like expertise, accuracy, and credibility (24–26). Trust in doctors is rooted in their professional qualifications and the credibility associated with their medical knowledge (27, 28). This trust is reinforced by their adherence to professional standards and high level of authority. In contrast, trust in family and friends is built over time through repeated interactions (29, 30), and the inherent trustworthiness attributed to familiar relationships (31, 32). Their advice, while not medically authoritative, is valued for its personal relevance, reassurance, and emotional support (33). While family and friends provide essential emotional support and online sources offer convenient access to a broad range of information, they lack the personalized touch, and the expert evidence-based knowledge provided by healthcare professionals. This expertise is unparalleled, making them the preferred source for reliable health information.

When it comes to online sources, the level of trust varies significantly based on the perceived credibility of the site (34, 35). For example, Wikipedia may be less trusted for health information due to its general nature and open editing model, which can lead to concerns about accuracy and specificity (36). In contrast, websites like WebMD are often viewed as more credible due to their focus on health information and perceived authority in the medical domain (37, 38). However, online platforms offer easy accessibility and immediacy, but may be as less trustworthy because they lack personalized advice, emotional support, and reassurance, making them seem contrived, less personally applicable, and less reassuring compared to interpersonal sources. Therefore, we hypothesize (H1) that individuals will display higher trust in human sources like doctors, friends/family, compared to online sources:

**H1:** Among various health information sources, human sources of information (e.g., friends/family, doctors) are rated as the most trusted by people.

The level of trust that individuals have in certain sources can vary depending on individual circumstances, such as symptom intensity or disease type. An individual’s experience with a particular illness may elicit levels of concern based on how severe the symptoms of the disease may be, which may in turn influence their information trust and consumption of that information (39). We will further explore how these key attributes of health conditions (i.e., indicators of disease severity) may influence trust in various sources. A study conducted during COVID-19 pandemic found that as symptom intensity increases, individuals tend to prefer trusting information from highly credible sources, such as the doctors (40). This underscores the role of doctor as the primary trust source for patients, particularly when symptoms are severe and precise information is crucial (41). Moreover, higher symptom intensity often correlates with a greater perceived risk (42, 43), leading individuals to rely on sources that offer professional expertise and authoritative guidance (44). In such cases, doctors, with their access to complete medical histories as well as their professional training, are more likely to be trusted for high-intensity symptoms that require precise and tailored medical advice.

While previous research has shown that individuals with high symptom intensity tend to seek out comprehensive information, and the satisfaction of the information received is related to the accuracy and reliability of medical advice (13, 45). For instance, friends and family may offer advice based on their own experiences and knowledge of an individual’s life and symptoms (46). However, the lack of formal medical training and limited depth of medical insights from these sources cannot replace the guidance from doctors (47). In cases of milder health conditions with lower perceived intensity and severity, patients may be more likely to consult less formal sources, such as online platforms (48). For instance, social networking sites (SNSs) like Twitter are more likely to be used by patients with less severe or intense health conditions for factual tasks (49). In these scenarios, the information might not necessarily have to be of the highest medical precision, as the perceived risks and urgency are lower (50). Individuals may consider information from these sources as ‘good enough’ for their needs, balancing the perceived credibility with the convenience and accessibility of the information (51, 52).

In general, doctors, different from family, friends, and online platforms, own a unique combination of professional training and authority in prescribing effective treatment protocols (53), which enables them to tailor recommendations not just to symptoms, but to the broader context of the individual’s health including lab data, nuanced understanding of disease mechanisms, progression, and evidence-based treatment options including medication interactions (54, 55). However, considering the varied levels of symptom intensity, the trust that people place in various information sources is likely to differ. Therefore, we hypothesize that (H2) people are more inclined to trust doctors over other sources if they perceive symptom intensity as high.

**H2:** Symptom intensity and information source will interact, such that when symptom intensity is high (versus low), people are more likely to place their trust in doctors over other sources.

Building on our understanding of how general trust in health information sources varies with symptom intensity, we will also investigate the impact of disease type on trust levels. This exploration will examine how the nature of diseases, whether chronic or acute, influences decisions on where to seek reliable health information. Previous research indicates that the management between acute and chronic diseases can be significantly different, which affects how people seek, process, and utilize health information and where they place their trust (56–58). Acute diseases such as influenza or COVID-19 are often sudden and severe but usually last a short time. In these cases, individuals may urgently seek immediate, specific, and actionable information (59). This urgency may lead people to broader and easier access to sources for quick answers. However, consulting with healthcare professionals, which can offer more personalized and comprehensive advice, often involves scheduling appointments or facing wait times, contrasting with the immediate access provided by less formal sources. As a result, those with acute ailments may view both online and interpersonal sources as sufficient for their short-term health information purposes.

In contrast, chronic diseases like diabetes or cancer often last more than a year or even require lifelong management (60). Such individuals with chronic diseases need to continuously engage in information-seeking to better understand and manage their condition, explore treatment options, and further adjust their lifestyle (61). Previous research suggests that for chronic diseases, trust in healthcare professionals becomes increasingly important, as the complexity and ongoing nature of chronic diseases requires a comprehensive treatment plan that effectively manages people’ health conditions with relevant knowledge, continuous support, and tailored advice from professional caregivers (62, 63).

While acute diseases often prompt individuals to seek immediate, less specialized information due to the urgency of their conditions, the complex nature of chronic diseases necessitates continuous and expertly guided management. Based on this understanding, we hypothesize that (H3) people with chronic diseases are more inclined to trust doctors over other sources.

**H3:** Disease type and information source will interact, such that people with chronic (versus acute) diseases are more inclined to place their trust in doctors as opposed to other information sources.

Recognizing the critical role of health conditions in influencing patients’ choices of health information, our study carefully considers how trust is measured. In this study, we examine two dimensions of trust: cognitive trust and behavioral trust. Cognitive trust refers to the individual’s belief in the reliability and dependability of an information source (64, 65). On the other hand, behavioral trust is established through the actions taken based on the information from the trusted source, which may not always align with cognitive trust due to the complex factors influencing decision-making (66, 67). Previous studies have indicated that a straightforward combination of cognitive and behavioral trust could not accurately predict people’s behaviors (68, 69). This inconsistency highlights the complexity of how trust influences health-related behaviors and suggests differences between one’s beliefs in the information source and their subsequent actions. Through an exploratory approach, this research can uncover whether there is a consistent relationship between people’ cognitive and behavioral trust in the context of health information.

Given these complex dynamics, our study investigates the interplay between disease type, symptom intensity, and various information sources to enhance our understanding of how people place trust in information sources under different health conditions. In an era where information is easily accessible from multiple sources, this research aims to provide crucial insights into people’ health information seeking behaviors and decision-making strategies. This will inform healthcare practice and patient education and further enhance our ability to design more effective and trustworthy HISs.

## 3 Materials and methods

In this section, we outline the study procedures used to examine patients’ trust in various health information sources under different health conditions. We first describe our data collection approach and the sample included in our analysis. Next, we detail the design and measures implemented to address our research questions. Finally, we present the manipulation check conducted prior to testing our hypotheses, ensuring the effectiveness and robustness of our methods.

### 3.1 Sample and apparatus

A total of 286 participants from a large US college located in the midwest were recruited for this study. The experiment was administered as an online survey using the web-based platform, Qualtrics. Participants were recruited through email invitations that directed them to the online survey. This study received ethical approval from the Institutional Review Board (IRB) of University of Nebraska, under IRB Protocol #0566-22-EP. Consent was obtained electronically at the beginning of the survey, where participants were informed about the study’s purpose, their voluntary participation, confidentiality measures, and their right to withdraw at any time without penalty. While demographic information was not the main focus of our study, we included it in Appendix 1 for completeness and to support any further analyses that might explore correlations with trust in health information sources.

In the survey, each participant was given one vignette (acute and chronic diseases) with randomly assigned symptom intensity (low or high intensity) and information sources (doctor, friends/family, Wikipedia, or WebMD). Specifically, the vignette presented a randomly selected scenario that contained an individual’s health condition with different degrees of severity. The information sources then provided a general recommendation based on the health conditions described in the scenario. The pilot study with a small subset of participants from a similar demographic as our main study were conducted to validate these vignettes. Feedback from this group was used to ensure that the scenarios were realistic and understandable. After filtering out responses with missing values and excluding 15 data from the pilot phase, we finalized 243 complete results. When considering the distribution based on disease types, we had 107 participants with acute diseases and 136 with chronic diseases. As for the sources of recommendations, there were 57 from WebMD, 55 from Wikipedia, 72 from close friends or family members, and 59 from doctors. Lastly, in terms of symptom intensity, 122 participants were classified as high intensity, and 121 as low intensity, shown in Table 1.

**Table 1:** Description of data.

Variables	Description	Levels and numbers (total: 243)
Disease type	Chronic or acute	Chronic (136) or Acute (107) disease
Symptom intensity	Symptom intensity of the disease	Low (121) or high (122) intensity
Information sources	The information source that provides the recommendation	Doctor (59), Friend/Family (72), Wikipedia (55), WebMD (57)

### 3.2 Design and measures

We utilized a 2 × 2 × 4 between-subject design, factoring in disease type (Acute vs. Chronic), intensity level (Low vs. High), and recommendation sources (Doctor, Friends/Family, Wikipedia, WebMD). This design allowed for the investigation of how different factors interact and influence trust in health information sources. The variables of disease type (acute vs. chronic) and symptom intensity (high vs. low) were selected to explore how trust dynamics vary across two categories (i.e., disease type and symptom intensity) of disease severity (e.g., situations requiring immediate versus ongoing health management). The inclusion of diverse information sources—from professional (doctors) to informal (family/friends) and online platforms (WebMD and Wikipedia)—provided a test of trust across different but commonly used health information channels. Additionally, the between-subject design was crucial for avoiding carryover effects, ensuring that each participant’s response was influenced only by their specific scenario without the interference from exposure to multiple conditions. Furthermore, a manipulation check was used to ensure that the information was perceived by participants as we intended and designed. Finally, the randomization process resulted in varied numbers of participants for each information source and disease type, reflecting the diversity of real-world conditions. By ensuring that each group has a sufficient sample size for statistical analysis, this design helps to minimize biases and accurately reflects the spectrum of health information-seeking behaviors.

In the survey, participants were randomly allocated to either an acute or chronic disease associated with one of four severity-based scenarios (such as acute with high intensity, acute with low intensity, chronic with high intensity, chronic with low intensity) and asked to read received symptoms for a medical condition at varying degrees of intensity. For example, the scenario included a fake name such as “chronitis” (“acutitis”) was given as a type of chronic (acute) disease with specific symptoms from real world diseases such as diabetes:

You were recently diagnosed with **Chronitis**, a chronic disease. Prior to this, your health was in a state that you believe to be [**poor**], and others would agree.

**[However, since being diagnosed, you have been constantly feeling weak both physically and mentally. You also cough sometimes, have muscle aches and pain, and a low-grade fever.]**

Within each of these scenarios, participants were assigned to one of four potential sources of healthcare recommendations (doctor, friends or family, Wikipedia, or WebMD). Additionally, we asked participants to rate their perceived disease severity. To control the influence of recommendation content, a non-specific recommendation was uniformly presented to all participants across the various cases:

*Doctor/Friend or family/Wikipedia/WebMD (one of the four recommendation sources) has recommended a course of action that involves taking a specific medication that can be acquired by the general public without a prescription.*

After being presented with the scenario and recommendation, participants were asked to decide whether they would accept the recommendation. This decision was measured by both behavioral and cognitive trust. Cognitive trust was specifically assessed using a 12-item scale originally developed for measuring trust in automation (70), which tests how much the participant trusts the recommendation (Appendix 2). Behavioral trust was assessed by binary response (yes or no) based on participants’ likelihood to follow the health advice given by each source. To answer our hypotheses, logistic regression, one-way analysis of variance (ANOVA), and two-way ANOVA were utilized to detect statistically significant differences in trust between sources under different health conditions. Specifically, ANOVA is suitable for our study as it allows for the analysis of main effects and interactions across groups, providing a comprehensive understanding of how different variables influence trust in health information sources. Similarly, logistic regression is appropriate given the categorical nature of certain response variables in our study, allowing for an assessment of how various factors predict binary decisions. Tukey’s Honestly Significant Difference (HSD) test was applied in post-hoc analyses to further explore specific pairwise differences. These methodological approaches allow us to understand the dynamic interplay of source type and disease intensity on trust, examining how different health information sources are trusted by participants across different health-related scenarios.

### 3.3 Manipulation check

To ensure that the intended manipulations have been successful in influencing participants’ perceived severity between different disease types and symptom intensity levels, we conducted a manipulation check before the tests for our hypotheses. Specifically, the perceived severity was compared between chronic/acute disease types and low and high symptom intensity with ANOVA test. The analysis revealed significant differences in perceived severity between chronic and acute diseases (*F*(1, 241) = 18.11, *p* < 0.001), with chronic diseases being associated with higher perceived severity levels. Similarly, significant differences were observed between low and high symptom intensity groups (*F*(1, 241) = 27.14, *p* < 0.001), indicating that participants perceived high symptom intensity as more severe compared to low symptom intensity. These findings validate the effectiveness of our experimental manipulations in influencing participants’ perceptions of disease severity including both disease types and symptom intensity.

## 4 Results

In this results section, we present the findings from our investigation into participants’ trust in various health information sources, directly correlating with our established hypotheses. Initially, we explored Hypothesis 1, which posits that human sources such as friends, family, and doctors are more trusted than other types of information sources, such as online sources. Next, we examined Hypothesis 2, which anticipates an interaction between symptom intensity and the choice of information source, predicting that higher symptom intensity leads to greater trust in doctors. Lastly, we assessed Hypothesis 3, proposing that the type of the disease (chronic versus acute) affects trust levels, with an expectation that individuals with chronic diseases show a stronger inclination to rely on doctors over other sources. Through logistic regression and ANOVA, complemented by post-hoc analyses, we elucidate how these factors influence trust, providing detailed insights into the specific interactions between health conditions and information sources.

The findings of Hypothesis 1 indicate that participants showed a significantly higher level of trust in human/interpersonal sources compared with online information sources. Specifically, one-way ANOVA results indicated that participants’ cognitive trust can be statistically significant between sources (*F*(3, 239) = 76.84, *p* < 0.001). With the results from post-hoc tests, Tukey’s HSD (Table 2), participants have significantly higher cognitive trust when the source is Doctor compared to WebMD (Mean Difference = 1.291, 95% CI: [0.952, 1.630], adjusted *p* < 0.001) and Wikipedia (Mean Difference = 1.536, 95% CI: [1.194, 1.878], adjusted *p* < 0.001). However, between interpersonal sources, Doctor and Close Friend/Family, it shows a small mean difference (0.080) with a high *p*-value (0.918), indicating no significant difference in cognitive trust scores between these two sources. Additionally, there is no significant difference between the cognitive trust scores for online information sources, Wikipedia and WebMD, as indicated by the adjusted *p*-value of 0.258. From the results of logistic regression (Table 3), behavioral trust was significantly higher when the source is Doctor compared to WebMD (Odds Ratio (OR) = 0.042, *p*-value = 0.009), Wikipedia (OR = 0.024, *p*-value = 0.002), and a close friend or family member (OR = 0.033, *p*-value = 0.0027).

**Table 2:** Post-hoc tests of one-way ANOVA for cognitive trust.

Comparison	Mean difference	95% CI	Adjusted *p*-value
Doctor vs. WebMD	1.291	[0.952, 1.630]	<0.001*
Doctor vs. Wikipedia	1.536	[1.194, 1.878]	<0.001*
Doctor vs. Close friend/family	0.080	[−0.241, 0.400]	0.918
Wikipedia vs. WebMD	−0.245	[−0.590, 0.100]	0.258
Close friend/family vs. WebMD	1.211	[0.887, 1.535]	<0.001*
Close friend/family vs. Wikipedia	1.456	[1.129, 1.783]	<0.001*

*N* = 243. *p < 0.05. **p < 0.01. ***p < 0.001.

**Table 3:** Logistic regression analysis for behavioral trust.

Variable	Coefficient (Estimate)	Odds Ratio	95% CI	*p*-value
Doctor (reference)	–	–	–	–
WebMD (compared to reference)	−3.178	0.042	[0.0041, 0.421]	0.0087**
Wikipedia (compared to reference)	−3.720	0.024	[0.0024, 0.248]	0.0017**
Friend/Family Member (compared to reference)	−3.401	0.033	[0.0036, 0.307]	0.0027**

*N* = 243. *p < 0.05. **p < 0.01. ***p < 0.001.

Based on the findings from both behavioral and cognitive trust analyses, Hypothesis 1 is supported. In terms of cognitive trust, human/interpersonal sources such as friends/ family, and doctors are more trusted than online sources. Additionally, no significant differences were observed between friends/family and doctors, indicating a comparable level of trust in both. Similarly, the cognitive trust between online sources, WebMD and Wikipedia, also showed no significant difference. The behavioral trust analysis reveals a distinct pattern that doctors are the most trusted source for health-related decision-making, followed closely by friends and family, while WebMD and Wikipedia are trusted less than these interpersonal sources.

Hypothesis 2 predicted that when the symptom intensity of disease is high, patients are more likely to trust doctors over other sources. The findings from the analyses indicate that participants showed a significantly higher level of trust in interpersonal sources compared to online information sources under high symptom intensity. However, results from a two-way ANOVA indicated that the interactions between information source and symptom intensity were not statistically significant overall (*F*(3, 235) = 0.256, *p* = 0.857), suggesting that differences in cognitive trust did not vary meaningfully between high and low disease intensity conditions for the specific sources tested (see Table 4). Similarly, logistic regression analyses showed no significant interaction between information source and symptom intensity as they relate to behavioral trust, such that symptom intensity did not influence the extent that people trusted different health information sources (see Table 5). In other words, participants reported feeling more willingness to take health advice in the Doctor group than other groups, but this effect did not differ by level of symptom intensity.

**Table 4:** Tests of two-way ANOVA for cognitive trust and disease intensity.

Comparison	*F* value	Pr (>*F*)	Mean difference	95% CI	Adjusted *p*-value
Main effects					
Source	77.325	<2e−16***			
WebMD vs. Doctor			−1.291	[−1.629, −0.953]	<0.001***
Wikipedia vs. Doctor			−1.536	[−1.877, −1.195]	<0.001***
Close friend/family vs. Doctor			−0.080	[−0.399, 0.240]	0.918
Wikipedia vs. WebMD			−0.245	[−0.589, 0.099]	0.256
Close friend/family vs. WebMD			1.211	[0.888, 1.534]	<0.001***
Close friend/family vs. Wikipedia			1.456	[1.130, 1.782]	<0.001***
Symptom intensity	4.754	0.030*			
Low vs. High			0.195	[0.017, 0.373]	0.032*
Interaction effects	0.256	0.857			
Source × symptom intensity (high)					
WebMD vs. Doctor			−1.273	[−1.830, −0.716]	<0.001***
Wikipedia vs. Doctor			−1.600	[−2.191, −1.009]	<0.001***
Close friend/family vs. Doctor			−0.035	[−0.595, 0.526]	0.999
Wikipedia vs. WebMD			−0.327	[−0.878, 0.224]	0.609
Close friend/family vs. WebMD			1.238	[0.720, 1.756]	<0.001***
Close friend/family vs. Wikipedia			1.565	[1.011, 2.120]	<0.001***
Source × symptom intensity (low)					
WebMD vs. Doctor			−1.239	[−1.831, −0.647]	<0.001***
Wikipedia vs. Doctor			−1.456	[−2.009, −0.903]	<0.001***
Close friend/family vs. Doctor			−0.109	[−0.621, 0.403]	0.998
Wikipedia vs. WebMD			−0.218	[−0.830, 0.395]	0.959
Close friend/family vs. WebMD			1.130	[0.553, 1.706]	<0.001***
Close friend/family vs. Wikipedia			1.347	[0.811, 1.883]	<0.001***

*N* = 243. *p < 0.05. **p < 0.01. ***p < 0.001.

**Table 5:** Behavioral trust of information sources and disease intensity.

Variable	Coefficient (Estimate)	Odds Ratio	95% CI	*p*-value
Main effects				
Doctor (reference)	–	–	–	–
WebMD (compared to reference)	−2.442	0.087	[0.022, 0.346]	<0.001***
Wikipedia (compared to reference)	−3.519	0.030	[0.006, 0.139]	<0.001***
Friend/Family Member (compared to reference)	−2.037	0.130	[0.033, 0.518]	0.004**
Symptom intensity				
High Symptom Intensity (reference)	–	–	–	–
Low Symptom Intensity	−0.056	0.945	[0.194, 4.594]	0.945
Interaction effects				
WebMD & low disease intensity	0.094	1.098	[0.160, 7.556]	0.924
Wikipedia & low disease intensity	0.621	1.861	[0.242, 14.292]	0.550
Friend/Family Member & low disease intensity	−0.156	0.856	[0.135, 5.413]	0.869

*N* = 243. *p < 0.05. **p < 0.01. ***p < 0.001.

Based on the analyses, Hypothesis 2 was not supported. The level of cognitive trust in doctors was significantly higher than in WebMD and Wikipedia but did not meaningfully differ from close friends or family, suggesting similar interpersonal trust. Behavioral trust analyses indicated that doctors are not only trusted more than WebMD and Wikipedia but also more than close friends and family. However, for the interaction effects, symptom intensity does not significantly alter the overall pattern of trust across different health information sources. Additionally—and although not formally part of our hypothesis tests—we found unexpectedly that the effect of symptom intensity on cognitive trust was significant (*F*(1, 235) = 4.754, *p* = 0.030), with low vs. high symptom intensity showing a small but significant difference (Mean Difference = 0.195, 95% CI: [0.017, 0.373], adjusted *p*-value = 0.032). That is, higher symptom intensity raises the level of trust that people place in their health information sources regardless of information source quality.

Hypothesis 3 predicted that people with chronic diseases are more likely to trust doctors over other sources. The findings from the two-way ANOVA indicate that the interaction between information source and disease type was not statistically significant (*F*(1, 235) = 0.475, *p* = 0.491), suggesting that cognitive trust did not vary meaningfully between acute and chronic disease types for the specific sources tested (see Table 6). Similarly, logistic regression analyses showed no significant interaction between information source and disease type as they relate to behavioral trust, such that disease types did not influence the extent that people trusted different health information sources (see Table 7).

**Table 6:** Tests of two-way ANOVA for cognitive trust and disease types.

Comparison	*F* value	Pr (>*F*)	Mean difference	95% CI	Adjusted *p*-value
Main effects					
Source	76.239	<2e-16***			
WebMD vs. Doctor			−1.291	[−1.631, −0.950]	<0.001***
Wikipedia vs. Doctor			−1.536	[−1.879, −1.192]	<0.001***
Close friend/family vs. Doctor			−0.080	[−0.401, 0.242]	0.919
Wikipedia vs. WebMD			−0.245	[−0.592, 0.101]	0.261
Close friend/family vs. WebMD			1.211	[0.886, 1.536]	<0.001***
Close friend/family vs. Wikipedia			1.456	[1.130, 1.785]	<0.001***
Disease type	0.475	0.491			
Chronic vs. Acute			0.063	[−0.118, 0.243]	0.494
Interaction effects	0.555	0.645			
Source × disease types (acute)					
WebMD vs. Doctor			−1.391	[−2.006, −0.777]	<0.001***
Wikipedia vs. Doctor			−1.687	[−2.266, −1.107]	<0.001***
Close friend/family vs. Doctor			−0.104	[−0.689, 0.480]	0.999
Wikipedia vs. WebMD			−0.295	[−0.900, 0.309]	0.810
Close friend/family vs. WebMD			1.287	[0.677, 1.897]	<0.001***
Close friend/family vs. Wikipedia			1.583	[1.010, 2.157]	<0.001***
Source × disease types (chronic)					
WebMD vs. Doctor			−1.217	[−1.751, −0.683]	<0.001***
Wikipedia vs. Doctor			−1.377	[−1.949, −0.805]	<0.001***
Close friend/family vs. Doctor			−0.057	[−0.560, 0.447]	0.999
Wikipedia vs. WebMD			−0.160	[−0.724, 0.405]	0.989
Close friend/family vs. WebMD			1.160	[0.665, 1.655]	<0.001***
Close friend/family vs. Wikipedia			1.320	[0.784, 1.856]	<0.001***

*N* = 243. *p < 0.05. **p < 0.01. ***p < 0.001.

**Table 7:** Behavioral trust of information sources and disease types.

Variable	Coefficient (Estimate)	Odds Ratio	95% CI	*p*-value
Main effects				
Doctor (reference)	–	–	–	–
WebMD (compared to reference)	−2.342	0.096	[0.022, 0.409]	<0.001***
Wikipedia (compared to reference)	−3.5185	0.030	[0.006, 0.179]	<0.001***
Friend/family member (compared to reference)	−2.0369	0.193	[0.049, 0.760]	0.023*
Disease type				
Acute disease (reference)	–	–	–	–
Chronic disease	−0.134	0.875	[0.156, 4.888]	0.870
Interaction effects				
WebMD & chronic disease	0.094	1.099	[0.220, 5.478]	0.932
Wikipedia & chronic disease	0.621	1.861	[0.243, 14.235]	0.643
Friend/family member & chronic disease	−0.156	0.856	[0.137, 5.334]	0.422

*N* = 243. *p < 0.05. **p < 0.01. ***p < 0.001.

Based on the analyses, Hypothesis 3 is not supported. The results show that patients’ cognitive trust in doctors was significantly higher than in WebMD and Wikipedia. However, cognitive trust levels between doctors and close friends or family did not significantly differ, indicating comparable interpersonal trust regardless of disease type. Behavioral trust analysis revealed that doctors are trusted significantly more than WebMD, Wikipedia, and close friends or family, highlighting a distinct preference for professional advice in behavioral decisions. Despite these distinctions, the type of disease, chronic vs. acute, did not significantly alter the general pattern of trust across different health information sources.

## 5 Discussion

In this study, we investigated trust levels in various health information sources among participants. Our key findings suggest a trust disposition favoring interpersonal sources of health information over online sources, particularly a significant preference for doctors over sources such as WebMD and Wikipedia. Additionally, our research uniquely distinguishes between cognitive and behavioral trust, offering a more nuanced analysis of how trust influences health-related decision-making. Our findings show that even though cognitive trust may not significantly differ among sources like doctors and close friends or family, behavioral trust is markedly higher for professional advice when making health-related decisions. This distinction is crucial as it suggests that while people may cognitively trust various sources to a similar extent, when it comes to actual health actions, they predominantly rely on medical professionals. The lack of significant differences in cognitive trust between doctors and close friends/family members, despite clear preference toward these individuals over online sources, suggests the strong importance of personal connections when it comes to healthcare decision-making. Furthermore, our study delves into the dynamics of trust under different health conditions of disease type and symptom intensity. The robustness of trust in professional medical advice, regardless of changes in symptom intensity and disease type, underscores the enduring nature of this trust across different health scenarios. When acting on a medical decision, participants showed increased behavioral trust in doctors over all other sources, including family and friends. These findings underscore the critical role of perceived expertise in addition to personal connection when it comes to behavioral trust (i.e., acting on the advice of experts). Our experimental vignette methodology allows us to observe the influence of simulated health conditions in a controlled environment, providing a clear view of trust dynamics without the confounding factors present in naturalistic settings.

Our finding, relating to people exhibiting lower behavioral trust toward WebMD, Wikipedia, and their close friends or family compared to doctors when it comes to decision-making, is in line with previous research suggesting a main reliance on healthcare professionals over alternative sources (5, 71). However, regarding cognitive trust, there are no significant differences observed between doctors and close friends/family members, nor between WebMD and Wikipedia. It appears that participants trust and acknowledge that both doctors and friends or family are sources of information and have valuable insights to share. For example, while doctors have professional expertise, close friends and family have personal understanding of an individual’s situation or circumstance. Meanwhile, when it comes to whom they would ultimately accept behavioral guidance from, participants chose to trust doctors or close friends/family members more than online sources such as WebMD or Wikipedia. These results suggest people’ preference for interpersonal sources over digital or online sources, aligning with other studies have also noted the importance of people’ interpersonal trust in health information, particularly in the relationship of patients and doctors (72, 73). Additionally, the observed preference for interpersonal sources over online platforms aligns with the broader psychological concept of social influence and trust dynamics (74). Social connections, based on familiarity, often have significant influence on individuals’ decision-making processes, particularly in the context of healthcare choices (75, 76). The discrepancy between cognitive and behavioral trust highlights the complexity of people’s decision-making processes and highlights the need for a deeper understanding of the factors influencing trust formation and utilization in healthcare settings. While people may trust different sources cognitively, they may still prioritize the advice and guidance of medical professionals when it comes to making healthcare decisions. However, the trusted relationship formed between patients and doctors should not be thought overly idealized. It requires continuous careful assessment to ensure success in information processing, medication adherence, health communication, and the overall delivery of healthcare.

The findings from this study suggest that symptom intensity plays a significant role in cognitive trust in health information sources. While higher symptom intensity generally raised trust in all sources, the most trusted source remained the doctor, followed closely by friends and family. This highlights the potential importance of emotional support and perceive expertise in high-stakes situations. The lack of significant interaction effects between source and symptom intensity suggests that while symptom severity increases trust, it does not differentially affect trust levels across information sources. Overall, the results of this study and those from studies looking at patient compliance suggest that even with high symptom intensity, concerns about patients overly trusting non-physician health sources can be tempered (77). The lack of significant interaction effects in our study suggests that while we manipulated symptom intensity and disease type, these factors did not significantly alter the established trust hierarchy between doctors and other sources. Participants maintained a strong trust in doctors across all scenarios, emphasizing the overall high regard for professional medical advice in health-related decision-making. Moreover, our experimental approach, which separates specific variables and directly tests their impacts on trust, helps to establish causality rather than mere correlation. For instance, while cognitive trust did not significantly differ between interpersonal sources like friends or family and doctors, behavioral trust shows significantly higher trust in doctors. This distinction between cognitive and behavioral trust reveals complex dynamics in how trust is established, mirroring the insights of McKnight and Chervany (78) regarding trust formation.

While individuals may cognitively trust personal connections similarly to medical professionals, when faced with critical health decisions, they place greater trust in professional advice. This behavior highlights the importance of considering both the accuracy and the context of information within online health information systems (HIS) (79). Online HISs are essential for equipping patients with the necessary knowledge to make empowered healthcare decisions (80). Meanwhile, it is also important to discuss why online sources, such as WebMD and Wikipedia, may be perceived as less trustworthy. Several contributing factors should be considered and investigated in future research, including a lack of transparency regarding the credentials of content creators, the prevalence of unverified claims, and limited regulatory oversight compared to traditional healthcare professionals (81–83). Unlike the interactions with friends or family and doctors, individuals may also distrust online sources due to the lack of personalized feedback (84, 85). Improving the trustworthiness of online health information sources may involve enhancing content credibility, ensuring transparency in sourcing, and providing real-time personalized advice to align better with patients’ needs. Future research could explore the impact of interactivity and information presentation on trust in these platforms, which may provide more insights into bridging the trust gap between professional advice and online health information. Additional direction in the future research could also conduct a comparative analysis to delineate the specific attributes that influence trust levels in different platforms such as WebMD and Wikipedia. This could include but not limited to examine the role of content credibility, user interface design, and the clarity of source disclosures. Understanding these factors can help in designing more effective HISs that better align with patients’ expectations and needs for trustworthy information.

Our study findings underscore the urgent need for significant enhancements to these systems to optimize patient empowerment and improve decision-making processes by providing relevant information and aligning with patients’ perceived trust. In light of this study’s findings, the importance of optimizing these systems as they are trusted less than interpersonal health information sources are critical. According to Eysenbach (79), online HIS must provide reliable information that aligns with the critical needs and contexts of patients, ensuring that professional guidance is readily available and prioritized in scenarios where decisions have significant health consequences. The following section will specifically explain the indications for the design of online HIS.

### 5.1 Design implications for online health information system (HIS)

Understanding how individuals’ trust behaviors change under different health conditions is crucial for designing effective online health platforms that support patients’ diverse needs. The results of our study hold significant implications for HIS and online platform design by elaborating on the dynamics of trust in health information sources, particularly in the context of varying disease intensity levels and disease types.

Specifically, people’ trust in doctors as the primary source of health information highlights the significance of professional medical expertise from the perspective of patients. Incorporating this understanding into the design of online HIS is crucial for ensuring that patients feel supported and empowered in their health information seeking experiences. One way to utilize patients’ trust in doctors within the platform design is by integrating features that facilitate continuous communication and collaboration between patients and healthcare providers. For example, implementing secure messaging systems or telehealth functionalities allows patients to directly connect with their doctors for personalized advice, guidance, and follow-up care. Additionally, by providing patients with doctor-verified information for education or case study during people’ health information seeking and processing experiences can facilitate the increases of health literacy by ensuring that patients receive accurate, reliable, and up-to-date medical information and support health communication between patients and doctors with clear, relevant, and consistent information. Therefore, our findings suggest several practical directions for HIS design:

1. Promoting credibility and effective presentation: Reflecting the study’s findings that participants trust professional advice more due to its perceived credibility, online HIS should prioritize effectively presenting and showcasing the credibility of their information. This might be achieved by prominently displaying the credentials of content creators, clearly labeling endorsed sources, and ensuring the sources are easily accessible. For instance, HIS can provide verification symbols next to content creators’ names to show verified medical professionals or endorsed health organizations. Such elements are likely to enhance patients’ trust by providing verifiable indicators that reflect the trustworthy attributes associated with professional medical advice.
2. Personalization and interactive engagement: Our findings reveal no significant differences in cognitive trust among interpersonal sources, potentially due to the personalized and interactive nature of the information they provide. To mimic this aspect, HIS should emphasize personalization and interactive engagement. Personalization ensures that the health content and recommendations are tailored to align with individual’s specific health condition, previous interactions, and preferences. This ensures that the information provided is directly relevant and applicable to each patient, potentially increasing the effectiveness and patients’ reliance on the system. By offering advice tailored to individual health conditions and enabling real-time interactions with healthcare professionals, HIS can address the key elements of interpersonal communication that build trust. For instance, HIS can allow patients to create personalized health profiles that can be used to deliver content tailored to their specific conditions or health concerns. Additionally, it can include a tool that allows patients to input symptoms or questions, which then guides them through an interactive assessment leading to tailored advice and information.

These features are designed to not only enhance the perceived credibility of the platform but also to significantly improve its practical utility for patients. By implementing these enhancements, HIS can evolve into integral tools for providing personalized information, managing health, making informed decisions, and facilitating real-time, interactive healthcare experiences. However, it is essential to conduct further studies and validations regarding the effectiveness of these credibility and interactivity measures. Continuous research will be critical in refining these features to ensure they meet the evolving needs of patients and effectively support the broader implementation of HIS platforms.

## 6 Limitation

Limitations of our study include a small sample size for additional three-way interactions such as among intensity, disease type, and information sources, as well as limited data size, geographic scope, and specificity in information source variables. Specifically, although we recognize the potential for intricate interactions among symptom intensity, disease type, and information sources, the current study design did not robustly support a detailed three-way interaction analysis without risking the clarity and focus of the research objectives. This limitation suggests a promising avenue for future research to explore these complex interactions with a design tailored for such depth. Additionally, while this study’s focus on college students from a single U.S. institution limits its generalizability, it is important to note that using college students is a common methodological approach due to the controlled environment and accessibility of the population (86). Further, the findings themselves apply to an otherwise technology-savvy population (i.e., college students might exhibit more comfort and trust in technology-mediated information than older populations), representing a conservative test of these phenomena. As such, use of a student sample allows greater theoretical generalizability to older populations than the reverse direction (i.e., older populations preferring interpersonal information over technology would be no surprise but leaves open the question of whether younger populations also show similar trust preferences) because it enables us to conclude that even college students––many of whom grew up using technology tools––trust interpersonal and expert sources (87). This approach allows for the establishment of initial insights that can be tested in more diverse populations in subsequent studies. Future research should aim to include a more diverse and representative sample of people nationwide to enhance the validity of the results. Additionally, while our study’s participants were presented with detailed vignettes, they were not actually experiencing the illnesses described. However, this type of study design is acceptable given the design of our study to minimize bias and simulate controlled conditions (88). Furthermore, additional variables such as the evaluation of individual health literacy should be included, which could influence people’ perceptions of health information and healthcare decision-making processes. Another limitation of our study is that we used discrete information source categories to test trust differences, rather than relying on more abstract variable constructs. Considering that interpersonal sources were generally most trusted, more research should be done to identify which elements of interpersonal interaction (e.g., interactivity, personalized feedback) are most attractive or valuable to patients’ health information search behaviors and acceptance. Finally, examining over-trust in information sources such as evaluating how people respond after receiving misinformation could provide valuable insights into patient-doctor relationships. Exploring this aspect could further explain the complexities of trust in healthcare professionals and its impact on people’s care.

## 7 Conclusion

In conclusion, our study provides explanations on the complicated dynamics of trust in health information sources among varying disease severity levels. By clarifying the changes in trust behaviors under different health conditions, we highlight the importance of tailored approaches in health informatics and online platform design. Our findings explore the role of disease type and symptom intensity in shaping trust preferences, suggesting patients’ consistent needs for communication and collaboration with healthcare providers. Moreover, the recognition of professional medical expertise as a factor of trust highlights the significance of integrating verified information and support mechanisms within online health platforms. This study also enriches the discussion on the design of health information systems (HISs), suggesting that these systems should prioritize features that enhance patients’ perceived trust and offer doctor-verified information. By understanding these associations of trust across diverse information sources, our study not only confirms existing theories but also expands our understanding, offering practical applications to enhance patients engagement and trust in HIS. Furthermore, the study suggests the need for future work to explore multiple dimensions of trust and the mechanisms behind trust phenomena in information sources, inspiring deeper investigation into patients’ trust in health information.

## Data Availability

The raw data supporting the conclusions of this article will be made available by the authors without undue reservation.
