# Supplementary material for: Exploring trust dynamics in health information systems: the impact of patients’ health conditions on information source preferences
Source: Front Public Health. 2024 Nov 22;12:1478502. doi: 10.3389/fpubh.2024.1478502 (PMC11622144; doi:10.3389/fpubh.2024.1478502)
Supplement: Supplementary file 1 [file Supplementary_file_1.docx]

**Appendix 1.** Description of Demographic Variables

| **Variable** | **Categories** | **N (Percentage)** |
| --- | --- | --- |
| **Gender** |  |  |
|  | Male  Female | 109 (44.86%)  134 (55.14%) |
| **Age** |  |  |
|  | 19-23  24-28  29-33  34-38  39-43  44-48  49-53  54+ | 154 (63.37%)  37 (15.23%)  18 (7.41%)  16 (6.58%)  6 (2.47%)  2 (0.82%)  4 (1.65%)  6 (2.47%) |
| **Marital Status** |  |  |
|  | Single  Married  Partnered  Divorced | 194 (79.84%)  30 (12.35%)  12 (4.94%)  7 (2.88%) |
| **Ethnicity** |  |  |
|  | Hispanic  Non-Hispanic White  Non-Hispanic Black  Non-Hispanic Asian  Non-Hispanic other | 22 (9.05%)  170 (69.96%)  13 (5.35%)  18 (7.41%)  20 (8.23%) |
| **Now Employed** |  |  |
|  | Yes  No | 208 (85.60%)  35 (14.40%) |

NOTE: N = 243.

**Appendix 2:** A 12-item scale for assessing cognitive trust using doctor as the referent information source.

| Please rate the extent to which you agree or disagree with the following statements: | | | | |
| --- | --- | --- | --- | --- |
| **1. Doctor is deceptive** | | | | |
| Strong disagree | Somewhat disagree | Neither agree nor disagree | Somewhat agree | Strongly agree |
| **2. Doctor behaves in an underhanded manner** | | | | |
| Strong disagree | Somewhat disagree | Neither agree nor disagree | Somewhat agree | Strongly agree |
| **3. I am suspicious of doctor's intent, action, or outputs** | | | | |
| Strong disagree | Somewhat disagree | Neither agree nor disagree | Somewhat agree | Strongly agree |
| **4. I am wary of doctor** | | | | |
| Strong disagree | Somewhat disagree | Neither agree nor disagree | Somewhat agree | Strongly agree |
| **5. Doctor’s actions will have a harmful or injurious outcome** | | | | |
| Strong disagree | Somewhat disagree | Neither agree nor disagree | Somewhat agree | Strongly agree |
| **6. I am confident in doctor** | | | | |
| Strong disagree | Somewhat disagree | Neither agree nor disagree | Somewhat agree | Strongly agree |
| **7. Doctor provides security** | | | | |
| Strong disagree | Somewhat disagree | Neither agree nor disagree | Somewhat agree | Strongly agree |
| **8. Doctor has integrity** | | | | |
| Strong disagree | Somewhat disagree | Neither agree nor disagree | Somewhat agree | Strongly agree |
| **9. Doctor is dependable** | | | | |
| Strong disagree | Somewhat disagree | Neither agree nor disagree | Somewhat agree | Strongly agree |
| **10. Doctor is reliable** | | | | |
| Strong disagree | Somewhat disagree | Neither agree nor disagree | Somewhat agree | Strongly agree |
| **11. I can trust doctor** | | | | |
| Strong disagree | Somewhat disagree | Neither agree nor disagree | Somewhat agree | Strongly agree |
| **12. I am familiar with doctor** | | | | |
| Strong disagree | Somewhat disagree | Neither agree nor disagree | Somewhat agree | Strongly agree |

NOTE: This scale includes both positively and negatively phrased items to assess cognitive trust. For analytical consistency, all item scores are converted to reflect trust in the same direction—higher scores consistently indicate greater trust, regardless of the item’s original phrasing.
